# Invasive Rhinocerebral Aspergillosis in a Patient With Poorly Controlled Diabetes Mellitus: A Clinical Challenge

**DOI:** 10.7759/cureus.97881

**Published:** 2025-11-26

**Authors:** Pedro Sá Almeida, Ana Maria Carvalho, Ana Rita G Magalhães, Maria J Brage, Beatriz T Exposito

**Affiliations:** 1 Internal Medicine, Unidade Local de Saúde de Trás-os-Montes e Alto Douro, Chaves, PRT; 2 Otorhinolaryngology, Unidade Local de Saúde de Trás-os-Montes e Alto Douro, Chaves, PRT

**Keywords:** amphotericin b-related nephrotoxicity, aspergillus fumigatus, diabetes mellitus, frontal sinusitis, hyperglycemia, immunocompromised host, invasive rhinocerebral aspergillosis, isavuconazole, voriconazole

## Abstract

Invasive rhinocerebral aspergillosis (IRCA) is a rare but devastating fungal infection that can progress silently until significant tissue destruction occurs. It most often affects immunocompromised individuals, particularly those with poorly controlled diabetes mellitus. Frontal sinus involvement is uncommon, yet it poses a high risk of intracranial spread and severe neurological complications.

We describe a 74-year-old woman with long-standing, poorly controlled type 2 diabetes mellitus (HbA1c 14.1%), referred for unstable glycemic control and subtle cognitive changes. Cranial imaging revealed left frontal sinus opacification with posterior wall erosion and intracranial extension. She was started on empirical amphotericin B and broad-spectrum antibiotics. Endoscopic sinus surgery confirmed posterior table dehiscence with dural exposure, and cultures identified *Aspergillus fumigatus*. Due to amphotericin B-related nephrotoxicity, antifungal therapy was transitioned to isavuconazole and later to oral voriconazole. With optimized insulin therapy and close multidisciplinary follow-up, the patient recovered fully, showing radiologic improvement one month after discharge.

This case highlights the strong association between poor glycemic control and invasive fungal infections. Frontal sinus involvement demands early recognition and aggressive treatment to prevent intracranial complications. Isavuconazole offered effective antifungal coverage with better renal safety in this setting.

IRCA can be subtle in its early stages, but rapidly becomes life-threatening once intracranial extension occurs. Early diagnosis, coordinated multidisciplinary care, and strict metabolic control are key to achieving favorable outcomes in these patients.

## Introduction

Invasive rhinocerebral aspergillosis (IRCA) is an aggressive fungal infection primarily affecting immunocompromised individuals, particularly those with poorly controlled diabetes mellitus. Chronic hyperglycemia leads to multifactorial immune dysfunction, impairing neutrophil chemotaxis and phagocytosis, and compromising mucosal epithelial integrity, thus creating a favorable environment for opportunistic pathogens such as *Aspergillus* spp. [1].

Although IRCA more commonly involves the ethmoid and maxillary sinuses, frontal sinus involvement, while less frequent, is particularly concerning due to its proximity to the anterior cranial fossa and the increased risk of intracranial extension, including brain abscesses, superior sagittal sinus thrombosis, and frontal osteomyelitis (Pott’s puffy tumor) [2,3]. This form of extension, albeit rare, is associated with high mortality and neurological morbidity, requiring early diagnosis and aggressive management.

Recent studies have highlighted the association between poorly controlled diabetes mellitus and aggressive forms of IRCA with frontal sinus and intracranial involvement. In a systematic review and meta-analysis by Kattar et al. (2021), type 2 diabetes mellitus was identified as a major risk factor for invasive disease, with intracranial extension present in up to 30% of severe cases [4]. Another multicenter observational study by Lai et al. (2020) demonstrated that frontal sinus involvement with central nervous system extension was significantly associated with increased mortality, with an odds ratio (OR) of 4.6 in diabetic patients [5].

Given the high mortality associated with IRCA with intracranial extension and the increasing prevalence of poorly controlled diabetes, early recognition of clinical and radiological signs suggestive of frontal sinus invasion is essential. Here, we present a case of invasive frontal sinus aspergillosis with intracranial extension in a patient with poorly controlled diabetes mellitus.

## Case presentation

We report the case of a 74-year-old female with a medical history of hypertension, dyslipidemia, depression, long-standing (18 years), poorly controlled (HbA1c of 14.1%) type 2 diabetes mellitus. The patient was being followed in primary care on basal insulin and oral antidiabetic agents, with documented end-organ damage (diabetic retinopathy). She was referred for internal medicine consultation for optimization of metabolic control; poor insulin administration technique, cognitive/behavioral complaints, and headache were noted. She denied fever, pain, or respiratory symptoms.

**Figure 1:**
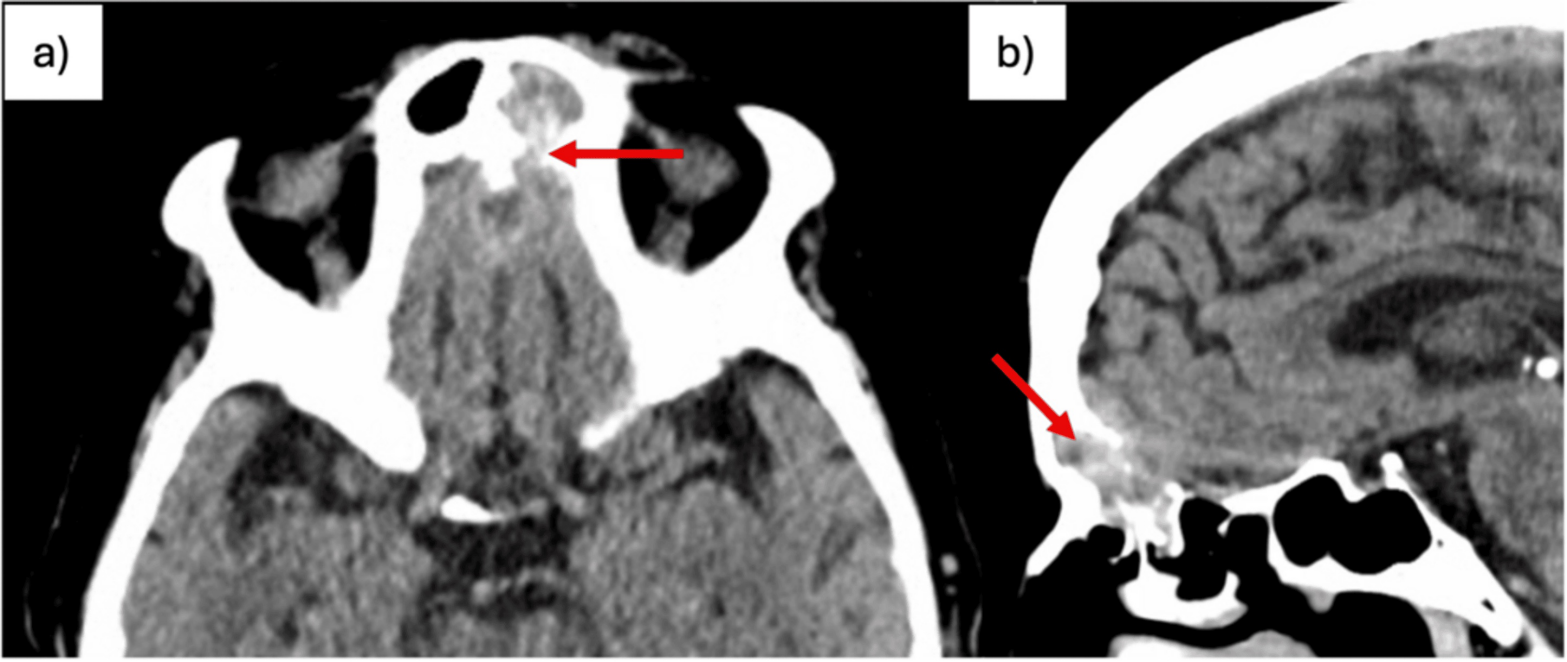
Cranial CT scan (a) Axial view; (b) Sagittal view. The left frontal sinus is filled with a soft-tissue component that, due to loss of the posterior bony contour, appears to extend anteriorly into the intracranial space along the midline (red arrows).

**Figure 2:**
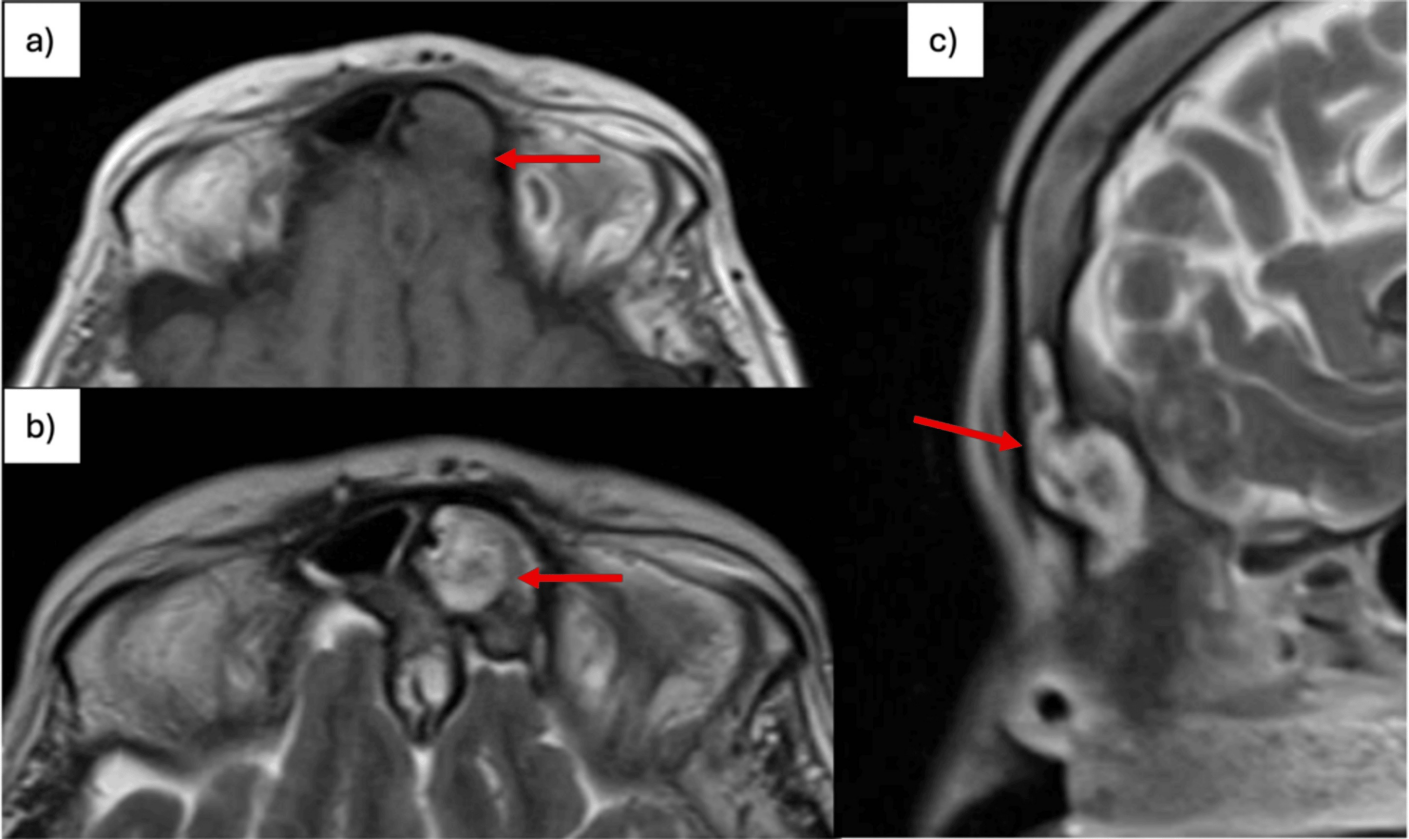
Cranial MRI (a) Axial T1-weighted image; (b) Axial T2-weighted image; (c) Sagittal T2-weighted image. The lesion is located in the anterior and inferior region of the interhemispheric falx, extending to the cribriform plates and the left frontal sinus, and measures approximately 13.5 mm in thickness and 62 mm along the craniocaudal axis (red arrows).

A cranial CT scan revealed a left frontal sinus filled by a tissue component, with posterior bony erosion suggesting anterior intracranial extension along the midline (Figure 1). Elective hospitalization was arranged for further investigation. Laboratory testing showed leukocytes of 6.68 ×10^3^/µL, C-reactive protein (CRP) of 1.52 mg/dL, blood glucose of 298 mg/dL, and a serum creatinine level (SCr) of 0.50 mg/dL. Cranial MRI showed the lesion located in the anterior inferior region of the interhemispheric fissure, extending to the cribriform plates and left frontal sinus, measuring 13.5 mm in thickness and 62 mm in maximum cranio-caudal axis (Figure 2).

According to the MRI report, an infectious collection was suspected despite the absence of fever. This led to blood cultures and cerebrospinal fluid analysis. Given the patient’s immunocompromised state from poorly controlled diabetes, empirical antifungal therapy with amphotericin B (1 mg/kg/day) was initiated. Following neurologic consultation and considering the possibility of a bacterial abscess/encephalitis, metronidazole (500 mg/day per os) and ceftriaxone (2 g/day IV) were added. Blood cultures grew *Escherichia coli* and *Streptococcus oralis*, sensitive to antibiotics, prompting continuation of antibacterial therapy and escalation of amphotericin B to 10 mg/kg/day (to achieve CNS penetration) after multidisciplinary discussion with the infectious disease department.

**Figure 3:**
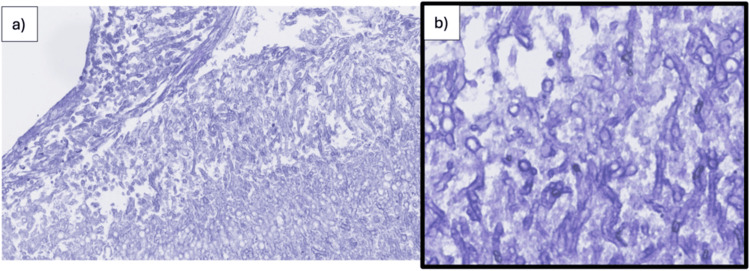
Histopathological images obtained during endoscopic sinus surgery (ESS) illustrating Aspergillus fumigatus. (a) Extensive area of fungal infiltration. (b) Characteristic hyaline, septate hyphae with acute-angle dichotomous branching typical of *A. fumigatus*, highlighted with Grocott’s methenamine silver (GMS) stain.

On day 7, the patient underwent endoscopic sinus surgery (ESS), with drainage of purulent material from the left frontal sinus sent for culture and histopathology. Posterior table dehiscence with dural exposure was noted, without cerebrospinal fluid leak. Six days postoperatively, cultures confirmed *Aspergillus fumigatus* (Figure 3).

By day 21, the patient developed nephrotoxicity from amphotericin B (peak SCr 2.30 mg/dL), prompting transition to intravenous isavuconazole (200 mg every eight hours for 48 hours, then 400 mg/day), cessation of metronidazole, and maintenance of ceftriaxone IV for six weeks. Insulin therapy and dietary education were optimized throughout hospitalization. Serial glucose measurements demonstrated a downward trend, decreasing from an admission value of 298 mg/dL to 75 mg/dL at discharge.

**Figure 4:**
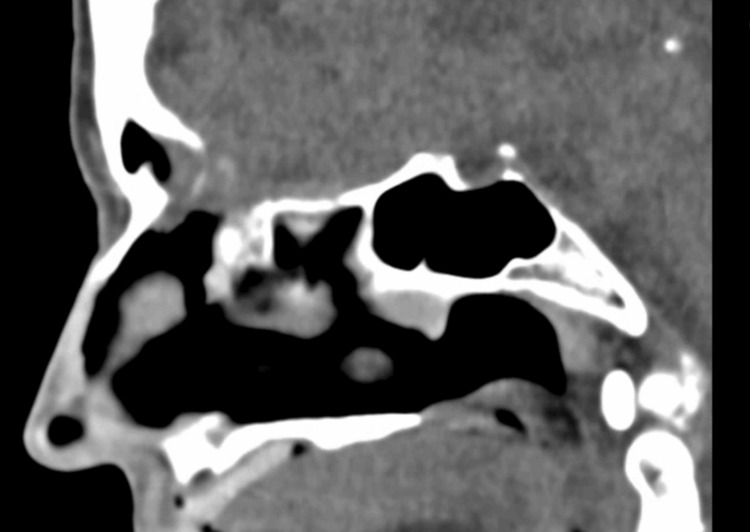
CT of the paranasal sinuses (CT-PNS) showing decreased filling of the left frontal sinus, without clear evidence of significant inflammatory recurrence. A persistent bony discontinuity is noted, with a potential communication to the anterior cranial fossa.

The patient was discharged after 50 days, asymptomatic and neurologically intact, continuing oral voriconazole (200 mg every 12 hours) to complete at least 12 weeks of antifungal therapy, along with insulin glargine 16 U/day, metformin 1000 mg + 850 mg/day, and dapagliflozin 10 mg/day. One-month follow-up showed the patient asymptomatic, compliant with therapy, and CT of the paranasal sinuses demonstrating reduced left frontal sinus filling without significant inflammatory recurrence (Figure 4).

## Discussion

IRCA is a rare but severe fungal infection, primarily affecting immunocompromised patients, particularly those with poorly controlled diabetes mellitus. In this case, marked hyperglycemia (HbA1c 14.1%) was associated with invasive fungal infection with intracranial extension, highlighting the key role of metabolic decompensation as a predisposing factor. Prolonged hyperglycemia impairs immune function, reducing neutrophil chemotaxis and phagocytosis, and induces microangiopathic changes compromising tissue oxygenation, creating an environment conducive to opportunistic fungal proliferation such as *A. fumigatus* [6].

Although IRCA may involve multiple paranasal sinuses, frontal sinus involvement, as observed here, is particularly concerning due to its proximity to the anterior cranial fossa and high risk of CNS dissemination [2-5,7]. Recent reports reinforce that frontal sinus involvement is associated with significantly increased mortality and neurological morbidity [5,7]. The present case illustrates this severity, as ESS revealed posterior table dehiscence and intracranial extension, despite the absence of clinical meningitis or established brain abscess. However, bacteremia with *E. coli *and *S. oralis* likely reflects translocation from the gastrointestinal and oral mucosa, underscoring her immunocompromised state and prompting consideration of systemic, including central nervous system, infection as a differential diagnosis, for which she was treated with targeted antibiotic therapy according to susceptibility testing.

Early diagnosis remains challenging, particularly in patients with atypical or subtle symptoms, such as mild headache and cognitive or behavioral complaints without fever, as illustrated by this case. Symptom paucity contributes to diagnostic delay, emphasizing the need for high suspicion in diabetic patients with destructive sinus lesions [4,8,9]. Imaging, particularly CT and MRI, plays a crucial role in identifying bone and intracranial involvement [3,10].

Management requires a multimodal approach combining systemic antifungal therapy and early surgical intervention. Recent evidence supports the combination of ESS with systemic antifungals as associated with improved outcomes and lower mortality [6,7,10]. In this case, initial amphotericin B, later switched to isavuconazole due to renal toxicity, extended with ESS for drainage, led to favorable outcomes. Isavuconazole was appropriate due to efficacy against *Aspergillus* spp. and better renal safety profile compared to amphotericin B [8,10].

Glycemic control is a critical component of management, as correction of hyperglycemia-induced immune dysfunction improves antifungal response and reduces recurrence risk [8,11]. In this case, insulin optimization and patient education were essential for clinical stabilization.

## Conclusions

Although rare, IRCA with intracranial extension underscores the need for clinical suspicion in diabetic patients presenting with nonspecific symptoms and destructive sinus lesions, especially in the frontal sinus. Early recognition, targeted antifungal therapy, and appropriate surgical intervention are pivotal for prognosis. This case highlights poorly controlled diabetes as a key predisposing factor, the importance of multidisciplinary management, and the necessity of early diagnosis to prevent potentially fatal intracranial complications.
